# Phagocyte Transcriptomic Analysis Reveals Focal Adhesion Kinase (FAK) and Heparan Sulfate Proteoglycans (HSPGs) as Major Regulators in Anti-bacterial Defense of *Crassostrea hongkongensis*

**DOI:** 10.3389/fimmu.2020.00416

**Published:** 2020-03-20

**Authors:** Yue Lin, Fan Mao, Nai-Kei Wong, Xiangyu Zhang, Kunna Liu, Minwei Huang, Haitao Ma, Zhiming Xiang, Jun Li, Shu Xiao, Yang Zhang, Ziniu Yu

**Affiliations:** ^1^CAS Key Laboratory of Tropical Marine Bio-resources and Ecology, Guangdong Provincial Key Laboratory of Applied Marine Biology, South China Sea Institute of Oceanology, Chinese Academy of Science, Guangzhou, China; ^2^University of Chinese Academy of Sciences, Beijing, China; ^3^Innovation Academy of South China Sea Ecology and Environmental Engineering (ISEE), Chinese Academy of Sciences, Guangzhou, China; ^4^Southern Marine Science and Engineering Guangdong Laboratory, Guangzhou, China; ^5^National Clinical Research Center for Infectious Diseases, Shenzhen Third People's Hospital, The Second Hospital Affiliated to Southern University of Science and Technology, Shenzhen, China

**Keywords:** phagocytes, heparan sulfate proteoglycans (HSPGs), focal adhesion kinase (FAK), transcriptome, *Crassostrea hongkongensis*

## Abstract

Invertebrates generally lack adaptive immunity and compensate for this with highly efficient innate immune machineries such as phagocytosis by hemocytes to eradicate invading pathogens. However, how extrinsically cued hemocytes marshal internal signals to accomplish phagocytosis is not yet fully understood. To this end, we established a facile magnetic cell sorting method to enrich professional phagocytes from hemocytes of the Hong Kong oyster (*Crassostrea hongkongensis*), an ecologically and commercially valuable marine invertebrate. Transcriptomic analysis on presorted cells shows that phagocytes maintain a remarkable array of differentially expressed genes that distinguish them from non-phagocytes, including 352 significantly upregulated genes and 479 downregulated genes. Pathway annotations reveal that focal adhesion and extracellular matrix–receptor interactions were the most conspicuously enriched pathways in phagocytes. Phagocytosis rate dramatically declined in the presence of an FAK inhibitor, confirming importance of the focal adhesion pathway in regulating phagocytosis. In addition, we also found that heparan sulfate proteoglycan (HSPG) families were lineage-specifically expanded in *C. hongkongensis* and abundantly expressed in phagocytes. Efficiency of phagocytosis and hemocytes aggregation was markedly reduced upon blockage of endogenous synthesis of HSPGs, thus implicating these proteins as key surface receptors in pathogen recognition and initiation of phagocytosis.

## Introduction

Phagocytes are crucial executors in innate host defense against invading microbial pathogens, including bacteria and fungi (1, 2). In mammals, neutrophils and macrophage constitute the bulk of these frontline defenders mediating diverse immunological processes including recognition, engulfment, and elimination of microbes (3–5). Impairment of phagocytic functions is often associated with microbial infections and could bring adverse consequences to pathogen replication, immune evasion, and host mortality (6). In teleosts, B lymphocytes were demonstrated to possess potent phagocytic and bactericidal capacities, implying incomplete hemocytopoiesis in the lower vertebrates (7, 8). In contrast, invertebrates have a simple but robust innate immune system to cope with dynamically evolving immune challenges (9, 10). Emerging evidences suggest that circulating hemocytes are indispensable to innate immune response, nutrition, wound healing, detoxification, and even shell mineralization (11, 12). Essentially, surveillance and elimination of pathogens depend heavily on phagocytic capacities of hemocytes, whose efficiency in containing and killing pathogens is intimately tied to disease resistance of individual hosts (13).

Over the past decade, substantial progress has been made on the molecular mechanisms underlying aspects of phagocyte immunity, such as pathogen recognition, phagocytic degradation, and immune evasion in mammals (14, 15). To illustrate, phagocytes can migrate toward infection sites through chemotaxis. Several chemokine receptors, such as C-X-C chemokine receptors and G protein–coupled receptors, are involved in sensing chemotactic ligands and initiating signaling transduction in host cells (16, 17). Consequently, pathogen-recognition receptors (PRRs) on phagocyte membranes recognize specific pathogen-associated molecular patterns and trigger off events leading to phagocytosis, phagosome maturation, and degradation of pathogen components (18). Generally, the Toll-like receptor, C-type lectin receptor, and NOD-like receptor are engaged as the principal phagocyte PRRs involved in initiation of an immune response (19–22). Furthermore, phagosome maturation is mediated by Rab GTPases, which drive the formation of phagolysosomes and lysosomal fusion to enable antimicrobial activities via toxic oxidants and proteolytic enzymes (23). However, despite extensive investigation on the molecular basis of mammalian phagocytic functions, systematic and comprehensive studies on immune processes in invertebrate phagocytes are still wanting.

Recent evidence shows that efficiency of phagocytosis is varied in different lineages (24, 25). Among them, filter-feeding species such as bivalves have the most efficient phagocytes (26, 27), providing an excellent model for investigating phagocyte immunity in lower invertebrates. Notably, the Hong Kong oyster (*Crassostrea hongkongensis*) is an edible bivalve species endemic to estuarine and coastal regions of the South China Sea, with an aquacultural history of more than 700 years (28). As a sessile bivalve species, *C. hongkongensis* lives by filtering seawater and is prone to pathogenic infections due to prevalence of microbes in the estuarine regions (29). Therefore, the oyster has evolved an efficient host defense system with high phagocytic activities to safeguard its survival within ecologically dynamic environments (30). Recently, advances in omics studies on the evolutionarily close *Crassostrea gigas* have demonstrated that lysosomal protease cathepsin L is one of the key contributors to pathogen killing in hemocytes (31). However, the other important events including pathogen recognition and activation of signaling pathways in phagocytes remain underexamined in oysters. In this study, *C. hongkongensis* phagocytes were systematically isolated by means of magnetic latex beads for cell sorting, and subsequent transcriptomic analysis provided a fuller picture on the molecular basis of phagocyte-dependent host defense. To highlight, the heparan sulfate proteoglycans (HSPGs) family was lineage-specifically expanded and enriched in expression in *C. hongkongensis* phagocytes, implying a crucial role for such surface receptors in bacterial recognition and phagocytosis initiation. In addition, we also found that focal adhesion kinase (FAK) signaling is a highly active process subserving key phagocytic functions including productive phagocytosis in oysters.

## Materials and Methods

### Animal Culture and Hemocyte Preparation

*Crassostrea hongkongensis* specimens consisted of 2-year-old healthy individuals with an average weight of 100 g and a shell height of 10.00 ± 0.05 cm. All samples were collected from a local breeding farm in Zhanjiang, China. Oysters were cultivated in aerated sand-filtered seawater for at least 1 week before experiments, and the culture was maintained at 22°C. Oysters were fed with *Tetraselmis suecica* and *Isochrysis galbana* every other day for 1 week prior to use. All experimental manipulations were performed in accordance with local guidelines on care and use of laboratory animals. Oyster hemolymph was extracted from cardiocoelom of the oysters by using a medical-grade syringe (0.45 × 15.5 mm) and hemolymph from each individual counted toward one sample. Samples were stored on ice and added to an equal volume of marine anticoagulant (MAC1; 0.1 M glucose, 15 mM trisodium citrate, 13 mM citric acid, 10 mM EDTA, 0.45 M NaCl, pH 7.0) to prevent coagulation. Physiological status of hemocytes was assessed under a light microscope (EVOS FL) to determine their suitability for subsequent experiments.

### Cell Sorting Assay With Magnetic Beads

Thirty-six oysters were randomly divided into groups in triplicates. Hemocytes in suspension were gathered as ~1 mL hemolymph per oyster into a 15 mL centrifuge tube (Corning, New York, USA). Cells were incubated with glucan coated magnetic beads (Micromod, Rostock, Germany), which are made of an iron oxide core with a diameter of 1.5 μm, at a ratio of 50 (i.e., 50 beads per cell). After 30 min of incubation, cells were resuspended in 20 mM HEPES solution (Sangon Biotech, Shanghai, China). Cells that had engulfed magnetic beads were absorbed to the tube wall by a magnetic grate, whereas other cells remained in the liquid phase. Cells from the liquid phase were transferred into a separate tube for analysis of phagocytosis. Then, magnetically retained cells were washed three times with HEPES solution. Cells exhibiting phagocytic properties toward magnetic beads were collected as a sample of phagocytes. Subsequently, all hemocytes were harvested as a pellet by centrifugation at 300 g at 4°C for 10 min.

### Library Construction and RNA-seq

Total RNA was extracted from hemocytes without lymph by using TriZol reagent (Invitrogen, California, America) according to manufacturer's instructions. Cells were ground in liquid nitrogen in a 2-mL tube, followed by homogenization for 2 min. The homogenate was centrifuged for 5 min, at 12,000 g at 4°C. Then, the supernatant was mixed with 0.3 mL chloroform/isoamyl alcohol (24:1), which was equilibrated with gentle shaking for 15 s, followed by centrifugation at 12,000 g at 4°C for 10 min. After centrifugation, RNA retained in the upper aqueous phase was recovered and transferred into a new tube with as the supernatant to which was added an equal volume of isopropyl alcohol, followed by centrifugation at 12,000 g for 10 min at 4°C. Upon removal of the supernatant, RNA pellet was washed twice with 1 mL prechilled 75% ethanol. The mix was centrifuged at 12,000 g at 4°C for 5 min. Residual ethanol was discarded, followed by air drying of the pellet for 5 to 10 min in a biosafety cabinet. Finally, 25 to 100 μL of DEPC-treated water was added to dissolve RNA. Total RNA was assessed for quality and quantified by using NanoDrop 2000 and Agilent Technologies 2100 bioanalyzer (Thermo Fisher Scientific, Massachusetts, USA). For reverse transcription into cDNA, oligo(dT)-attached magnetic beads were used to purify mRNA and deplete rRNA. Purified mRNA was fragmented into small pieces with fragment buffer. First-strand cDNA was generated by using random N6 primer and hexamer-primed reverse transcription, followed by second-strand cDNA synthesis. Subsequently, cDNA fragments obtained from previous steps were amplified by polymerase chain reaction (PCR), and products were purified by Ampure XP beads (Beckman Coulter, California, USA) and eluted in EB solution. The final products were validated in an Agilent Technologies 2100 bioanalyzer for quality control. Double-stranded PCR products from previous steps were heated for denaturing and circularized by a splint oligo sequence to accomplish the final library. Single-strand circle DNA was formatted as the final library, which was amplified to make DNA nanoballs comprising more than 300 copies of one molecule. DNA nanoballs were loaded into the patterned nanoarray, and single-end 50 bases reads were generated on a BGI seq500 platform (BGI, Shenzhen, China). All raw data were submitted to the NCBI database with the accession number SRR10531303–SRR10531311.

### Bioinformatics Analysis

Based on algorithms of the software SOAPnuke, clean reads were isolated from raw data and saved in FASTQ format in preparation for quantitative analysis. These clean reads were mapped onto a *C. hongkongensis* transcriptome database by using Bowtie2 (32), and mapped reads subsequently were summarized and normalized to RPKM by means of the RESM software (33). In addition, R cor was used to calculate the Pearson correlation coefficients of the samples. Principal components analysis was performed by using princomp. Differentially expressed genes (DEGs) were tested for statistical significance by DEseq2 methods, based on negative binomial distribution with the following threshold settings: fold change ≥2.00 and adjusted *p* < 0.05 (34). Further, the genes were subjected to analyses with Gene Ontology (35) and KEGG Orthology (36). Gene Ontology functional enrichment was also performed by utilizing phyper of R and *p*-value. Finally, false discovery rate (FDR) was calculated for each *p*-value, and we defined FDR < 0.01 as significantly enriched (37). Amino acid sequences of HSPGs orthologs in the target species were obtained by homologous Blast with the NCBI database. The GenBank accession numbers corresponding to the HSPG sequences analyzed are as listed in Supplementary Table 4. A phylogenetic tree was constructed with Clustal Omega (https://www.ebi.ac.uk/Tools/msa/) by the neighbor-joining method and analyzed with Interactive Tree of Life program, iTOL (http://itol.embl.de/). Protein domains and signal peptides were predicted with Simple Modular Architecture Research Tool (SMART), version 4.0 (http://smart.embl-heidelberg.de/).

### Flow Cytometric Analysis

Approximately 10^5^ hemocytes were plated to six-well plates and supplemented with an equal volume of lymph and left for further culture for 20 min. Then, live *Escherichia coli* with a green fluorescent protein (GFP) plasmid was added into the wells to achieve an MOI (multiplicity of infection) of 50. After 30 min, the antibiotic gentamicin (50 μg/mL) was added to kill off non-engulfed bacteria outside host cells, for incubation for 10 min (38). Cells were then washed three times with 20 mM HEPES solution (Sangon Biotech, Shanghai, China). Finally, cells were resuspended to detect the GFP fluorescence intensity by Guava easyCyte 5HT flow cytometer (Guava Technologies, California, USA). Cells were selected from the cyclized gate to remove cell debris. And all the conditions were adjusted to the proper level. Following step collected 10,000 hemocytes and was analyzed by FlowJo-V10 software (New Jersey, USA), in which three biological repeats were guaranteed for each group, and one group of hemocytes without any treatment was set as negative control.

### Validation of Biological Effects via Pharmacological Inhibition of Proteins

The inhibitors served as the specific blocker to suppress the function of HSPGs and FAK. Heparin (MedChemExpress, New Jersey, USA) and chlorate (J&K Scientific, Beijing, China) were the common inhibitors for decreasing expression of HSPG, and PF-573228 (Selleck Chemicals, Houston, USA) did that for FAK. Experiment set three kinds of concentration gradients to treat the hemocytes; each group was disposed with the same amount of time. Focal adhesion kinase inhibitor solution (concentration: 0, 1, 5, 10 μM) was dissolved in 20 mM HEPES solution and incubated with hemocytes for 30 min. Chlorate as an HSPG specific inhibitor could work after 2-h treatment in hemocytes. Its concentration gradient was set for four groups, 0, 1, 5, and 10 mM, respectively. But heparin was diluted by HEPES solution for final concentrations, 0, 10, 20, and 40 μM, incubating with cells for 30 min. Following that the hemocyte was stimulated by the GFP fluorescent bacteria for 30 min, the data of fluorescent intensity from cells was acquired by flow cytometry to figure out the proper concentration and the effect of these three factors, when the experimental group was compared to the control.

### Confocal Microscopy

Prior to phagocytic experiment, hemocytes were cultured in Petri dish for 20 min. Afterward, the hemocytes were subject to different treatment with the three kinds of inhibitors at the proper concentrations, respectively, of which sodium chlorate is a metabolic inhibitor to prevent proper sulfation of HSPGs, whereas heparin is a glycosaminoglycan (GAG) competitively inhibiting tau binding to HSPGs. Then, removing the drug, the GFP fluorescence bacteria (the proportion with the cells 50:1) were added into the dish to stimulate the hemocytes starting phagocytosis. Half an hour later, the gentamicin solution was used to kill bacteria that were outside of cells; further washing buffer removed the extra bacteria. Then, the hemocytes were fixed for 20 min in 4% paraformaldehyde dissolved in phosphate-buffered saline. HEPES solution was used to wash the cells before the cell nucleus of hemocyte was stained by DAPI (Sigma, California, USA) excited by the violet (405 nm) laser line; the cytomembrane was fluorescent red by Dil (Beyotime, Shanghai, China) excited by the violet (549 nm) laser line. Confocal microscopy was performed to image the difference of these two groups compared to the control. After hemocytes were incubated with red fluorescent beads, confocal microscopy captured the pictures to illustrate the cellular morphology, and ImageJ (Maryland, USA) calculated the fluorescent intensity. For aggregation test, hemocytes were transferred into Petri dishes for 20 min. Then, heparin and chlorate made the HSPG loss activity after treatment; the cell nucleus was fluorescent blue by DAPI, and the cytomembrane was fluorescent red by Dil. Confocal microscopy took the images for analyzing the significant difference of aggregation among these data. The software ImageJ assisted in analyzing the image data.

### Statistical Analysis

Statistical analysis was performed with GraphPad Prism 8 (GraphPad Software Inc., San Diego, California, USA), and values were expressed as mean ± SD. Statistical significance of differences between the control and treated groups was determined by Student *t*-test or one-way analysis of variance. Differences determined at ^*^p < 0.05, ^**^p < 0.01 or ^***^p < 0.001 were considered significant.

## Results

### Isolation of Oyster Phagocytes by Magnetic Bead Sorting

**Figure 1:**
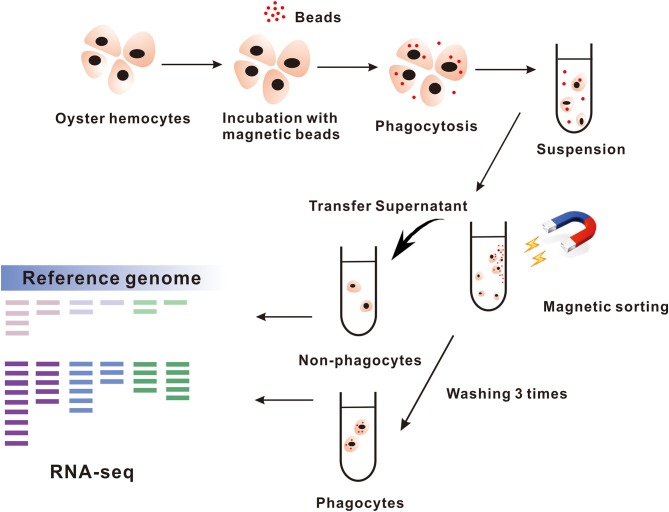
The process of magnetic beads sorting phagocytes. The hemocytes in *Crassostrea hongkongensis* are gathered for incubating with magnetic beads and then sorted by magnetic grate. The cells remaining in supernatant are the non-phagocytes. Transcriptome analyzes the samples of phagocytes and non-phagocytes.

A cell sorting assay based on magnetic beads was used to separate phagocytes and non-phagocytes from total hemocytes, in which magnetic-enriched cells were considered as phagocytes, and non-enriched cells were non-phagocytes (Figure 1). Our results showed that 87.6 to 91.7% of magnetically sorted phagocytes engulfed at least one magnetic beads, whereas only no more than 5% of non-phagocytes contained them (Supplementary Figure 1). Meanwhile, we also observed that the majority of the phagocytes were composed of granulocytes (82.8%) with hyalinocytes as a minority, which suggests that a small proportion of hyalinocytes could participate in phagocytic defense.

### Transcriptomic Analysis of Regulatory DEGs in Phagocytes

**Figure 2:**
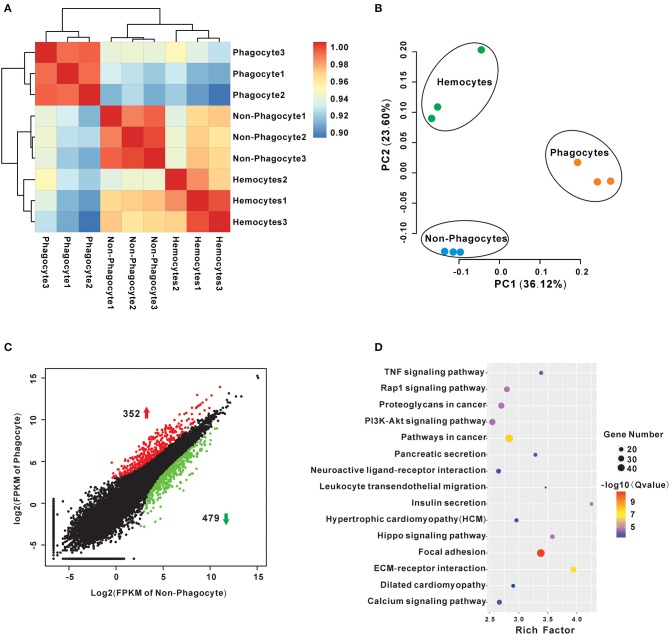
Transcriptome analysis of differences between *Crassostrea hongkongensis* phagocytes and non-phagocytes. **(A)** Transcript expression profiles of hemocytes were analyzed by means of RNA-seq quantification. The heatmap shows the square of correlation among phagocyte, non-phagocyte, and hemocyte group. Each sample contains three independent biological replicates. **(B)** Principal components analysis plots of gene expression for the three groups of hemocytes using the normalized transcriptome expression. Each group was represented by different-color dots: green for hemocytes, orange for phagocytes, and blue for non-phagocytes. **(C)** Identification of DEGs between phagocytes and non-phagocytes. The red dots show genes were significantly upregulated in phagocytes, whereas the green dots denote downregulated genes. **(D)** KEGG enrichment analysis of DEGs between phagocytes and non-phagocytes. The scatterplots illustrate the DEGs enriched in many functional pathways. The enrichment factor of this figure is the ratio of the DEGs number to that of total sample; the dot size denotes the number of DEGs with different colors that are according to the *q*-value range.

A series of RNA-seq libraries were constructed from phagocytes, non-phagocytes, and hemocytes, each group consisting of three biological replicates. Further sequencing and low-quality filtration, each one obtained about 23.8 million clean reads, which were mapped onto a Hong Kong oyster genome (unpublished data) as the reference. Percentages of total mapped reads approached 76.18–80.19%, whereas percentages of unique mapped reads were about 71.34–74.53% (Supplementary Materials). Pearson correlation analysis of total expression profiles reveals that there is a high correlation within each group, supporting the reliability of biological replicates (Figure 2A). Principal component analysis further reveals that phagocytes had distinct expression profiles vs. that of non-phagocytes (Figure 2B). Next, we pinpointed DEGs of phagocytes through comparisons with non-phagocytes. A total of 831 DEGs comprising 352 upregulated genes and 479 downregulated genes were found (Figure 2C, Supplementary Table 2). Remarkably, several signaling pathways, in particular, focal adhesion (48 genes, rich factor 3.38, *p* = 1.19 × 10^−13^), extracellular matrix (ECM)–receptor interaction (29 genes, rich factor 3.94, *p* = 2.93 × 10^−10^), PI3K-Akt signaling pathway (33 genes, rich factor 2.54, *p* = 8.86 × 10^−7^), and tumor necrosis factor signaling pathway (19 genes, rich factor 3.38, *p* = 3.67 × 10^−6^) were significantly enriched in professional phagocytes, suggesting the coordinated nature of these regulatory genes in oyster phagocytes (Figure 2D, Supplementary Table 3).

### Focal Adhesion Signaling Pathway Is Active in Phagocytes and Regulates Phagocytic Capacity

**Figure 3:**
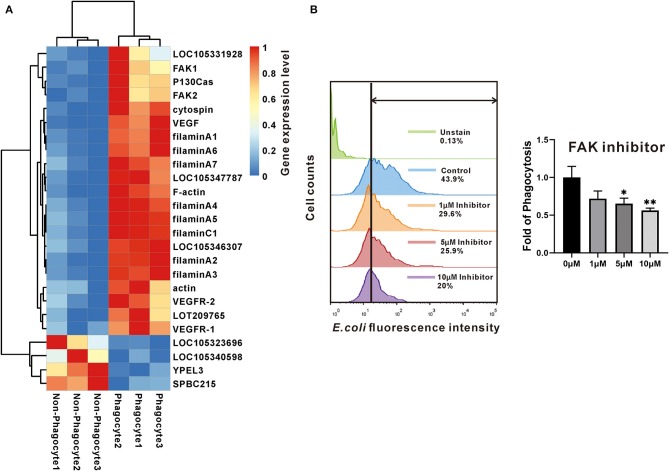
The expression and effect of FAK in phagocytes. **(A)** The heatmap chart used the changes in colors to show differences of the gene expression level between phagocytes and non-phagocytes. **(B)** The effect of FAK in phagocytosis. Flow cytometry determined the fluorescence intensity that hemocytes contained. Green shows the data of negative control; blue is the normal control; orange, red, and purple show the experimental samples that were treated with different concentrations of inhibitor. While the bar chart compares the fold of phagocytotic ability among the four groups that were under concentration gradient of FAK inhibitor. The bars indicated the fold of phagocytosis. The amount relative to the internal control is expressed as mean ± SD (*n* = 3). Significant differences relative to control were indicated (*P <0.05 and **P < 0.01).

Focal adhesion is one of significantly enriched signaling pathways with highest confidence values as mentioned previously. Indeed, 25 of DEGs were included, and 21 of them were significantly high expression in phagocytes, highlighting the active focal adhesion signaling pathway in oyster phagocytes (Figure 3A). Specifically, two of FAKs, one of P130Cas, and eight of filamins (seven of filamin A and one of filamin C) were identified, and they constituted the key components of this pathway (Figure 3A). Within this pathway, FAK is an indispensable member that orchestrates exogenous signaling to instruct cell behaviors. To validate whether FAK controls phagocytic capacities of oyster hemocytes, the FAK-specific inhibitor PF-573228 was used to block activation of FAK signaling. Results show that PF-573228 suppressed the phagocytic abilities of hemocytes in a dose-dependent manner, with a 0.34- and 0.44-fold change in phagocytic ability being elicited by the inhibitor at 5 and 10 μM, respectively (Figure 3B), implying a crucial role of FAK in phagocyte activation.

### HSPGs Are Lineage-Specifically Expanded and Abundantly Expressed in Phagocytes

**Figure 4:**
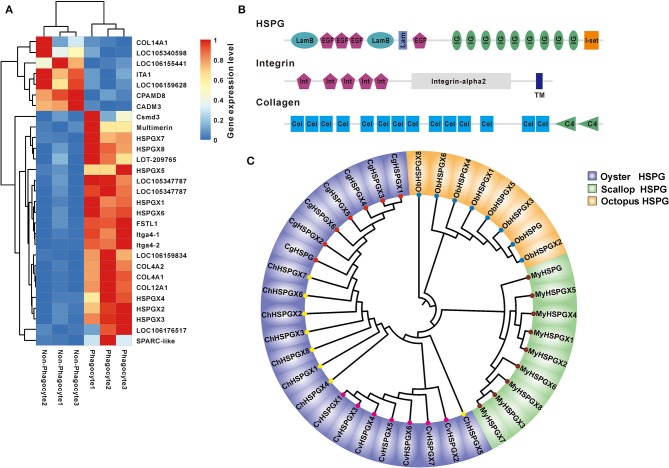
The expression and bioinformatics analysis of HSPGs. **(A)** The heatmap presents the expression level of ECM–receptor interaction in phagocytes and non-phagocytes. **(B)** The domain architecture of HSPG, integrin, and collagen. **(C)** Phylogenetic analysis of mollusk HSPGs, which include *Crassostrea gigas, Crassostrea virginica, Crassostrea hongkongensis, Mizuhopecten yessoensis*, and *Octopus bimaculoides*. The neighbor-joining phylogenetic tree was constructed with 1,000 replications of bootstrap.

Another significantly enriched pathway is an ECM–receptor interaction pathway with secondary high confidence value (2.93E-10). Heatmap showed that a total of 29 DEGs were involved in ECM–receptor interaction, and 75.86% (22/29) were dominantly expressed in the phagocytes, among which three main matrix proteins were found to be eight of HSPGs, two of integrins, and four of collagens (Figure 4A). Strikingly, the structure organization showed that HSPG contains massive tandem IG domain, suggesting its possible function in bacterial recognition and immune regulation (Figure 4B). Moreover, phylogenetical analysis indicated that members of HSPG family are clustered into lineage-specific clades, strongly implying lineage-specific expansion of HSPGs in mollusks (Figure 4C). Additionally, eight of HSPGs in *C. hongkongensis*, seven of HSPGs in *Crassostrea virginica*, and seven of HSPGs in *C. giags* were also clustered into species-specific clades except for *Ch*HSPGX5, suggesting that the HSPG family was recently expanded after oyster speciation (Figure 4C). Taken together, specific expansion and dominant expression of HSPGs in oyster phagocytes highlight its essential role in innate defense.

### Blockade of Phagocytosis and Aggregation by HSPG Inhibitors in Hemocytes

**Figure 5:**
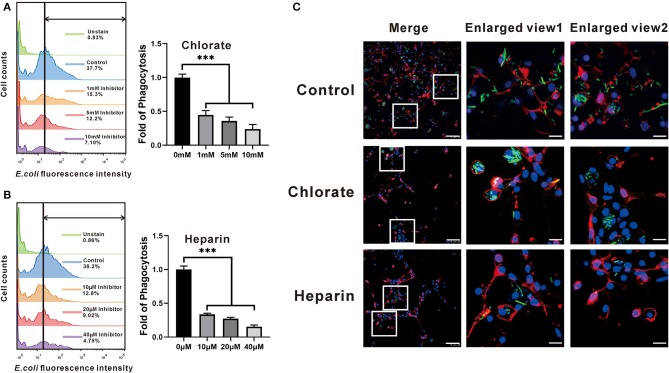
Comparison of the phagocytotic ability among the four groups that had different expressions of HSPG. The expression of HSPG was reduced by the specific inhibitors, chlorate **(A)** and heparin **(B)**. The changes of fluorescence intensity that hemocytes contained were detected by flow cytometry. And the bar charts compare the phagocytotic ability of different groups in fold. The bars indicate the fold of phagocytosis. The amount relative to the internal control is expressed as mean ± SD (*n* = 3). Significant differences relative to control were indicated (***P < 0.001). **(C)** The phagocytotic function of HSPG on the phagocytes. The figures show the phagocytotic changes of hemocytes that were captured by confocal microscopy. The DAPI represents the nuclear; the dye of Dil shows the position of cell membrane; the green fluorescence is on behalf of bacteria. The scale bar of enlarged views is 5 μM; the others are 25 μM.

**Figure 6:**
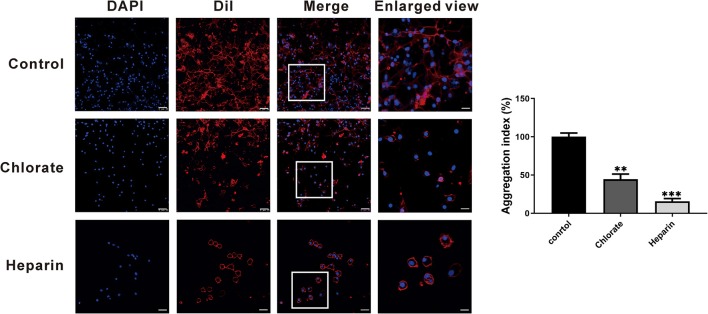
The agglutinative effect of HSPG on the phagocytes. The hemocytes were stained with DAPI and Dil to show the distribution of cells. The control group compares with other experimental groups that were treated with two kinds of specific HSPG inhibitors, chlorate and heparin. The concentrations used for chlorate and heparin were 1 mM and 10 μM. The scale bar of enlarged views is 5 μM; the others are 25 μM. The bar chart indicated the aggregation index. The amount relative to the internal control is expressed as mean ± SD (*n* = 3). Significant differences relative to control were indicated (**p < 0.001 and ***p < 0.001).

To examine the exact roles of HSPGs in oyster hemocytes, two chemical inhibitors, chlorate and heparin, were utilized to assess the effects of HSPGs on hemocyte function. Flow cytometry analysis shows that chlorate, an inhibitor for prevention of HSPGs sulfation, significantly decreased by 0.55- and 0.76-fold of phagocytic activities at concentrations of 1 to 10 mM (Figure 5A). Similarly, the other competitive inhibitor of HSPG, heparin, also demonstrated strongly suppressive effect on phagocytic ability of hemocytes, which obviously reduced 0.66- and 0.85-fold when treated at 10 to 40 μM (Figure 5B). Meanwhile, the inhibitory effects of chlorate and heparin on the phagocytic activities were confirmed by observations in confocal microscopy, where the concentrations used for chlorate and heparin were 1 mM and 10 μM, respectively (Figure 5C). Additionally, HSPG inhibitors also had clear effects on the aggregation of hemocytes. Compared to resting hemocytes, treatment with either chlorate (1 mM) or heparin (10 μM) resulted in a decrease of 55.6 and 84.2% aggregation of hemocytes, respectively (Figure 6).

## Discussion

Phagocytes in vertebrate or invertebrates are uniquely endowed with a powerful antimicrobial apparatus, which is characterized by efficient engulfment and subsequent destruction of invading pathogens during phagocytosis (39). In bivalves including *C. hongkongensis*, hemocytes populating the circulatory system make up an expert cell population dedicated to innate immune defense. Conventionally, these have been classified into at least two hemocyte subtypes, granulocytes and hyalinocytes, on grounds of morphology and function (40, 41). Although granulocytes are typically more active in phagocytic activities and reactive oxygen species production compared with hyalinocytes (42), growing evidence suggests that fractions of hyalinocytes also possess capacities for phagocytosis and clearing foreign particles or pathogens (43, 44). In all cases, elucidating the exact mechanisms of phagocytic defense necessitates reliable isolation of phagocytes from a complex mélange of hemocytes having an apparent continuum of differentiation status. To set out for this task, we first successfully isolated the phagocytes from oyster hemocytes by means of cell sorting with magnetic latex beads, which is an efficient and convenient isolation approach widely used in studies on neuronal and megakaryocytic cells (45, 46).

In transcriptomic analysis on phagocyte activation, two significantly enriched and functionally related major pathways, focal adhesion and ECM–receptor interaction, emerged with the highest statistical confidence. Characteristically, the ECM consists in part of secreted extracellular macromolecules, including collagen fibers, proteoglycans, and adhesive matrix proteins (47, 48). Consequently, ECM forms an essential microenvironment to provide a structurally and biochemically dynamic scaffold for surrounding cells, with vital regulatory roles such as cellular communication, cell migration, growth, and differentiation (49). Moreover, accumulating evidence shows that ECM function is instrumental to many facets of host immunity such as phagocytosis, aggregation, and endocytosis in both vertebrates and invertebrates (50–53). For instance, integrin-dependent phagocytosis has been identified in many invertebrate species, including shrimp (54), phylum Cnidaria (55), *Geodia cydonium* (56), *Mytilus trossulus* (57), and *C. gigas* (58). As a molecular pattern recognition receptor, integrin has been reported to mediate invasion of *Vibrio splendidus* LGP32 into hemocytes of *C. gigas*, illustrating the multilayered function of integrin in phagocyte promotion or pathogen invasion (38, 59). Indeed, high abundance of integrin was invariably observed in oyster phagocytes, consistent with its functional importance.

**Figure 7:**
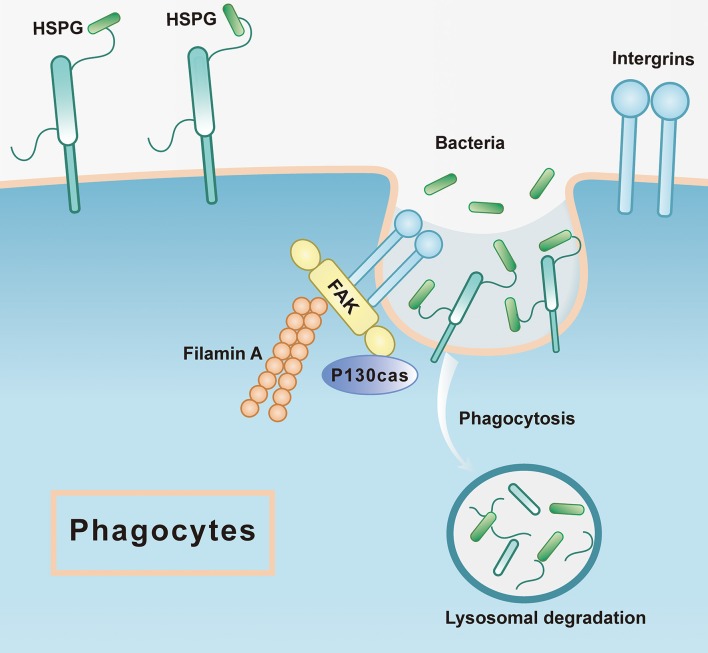
The mode pattern of FAK and HSPG in phagocytes. The HSPG works as the receptor that is located at the cell membrane to recognize the microorganism such as bacteria, which is the first step of phagocytosis, while the integrin could stimulate the downstream pathways where FAK is active to associate the other proteins such as filamin A, P130cas. Subsequently, the bacteria would be sent to lysosomal degradation.

Moreover, FAK, another significantly enriched pathway in phagocytes, has been recognized as a regulator centrally linking integrin signaling to cell response, including cytoskeleton remodeling, cell migration, and phagocytosis (60, 61). Two orthologs of FAKs are present in the transcriptomics of *C. hongkongensis*, both of which showed significantly high transcriptional expression in activated phagocytes. Functional validation demonstrates that phagocytic rate dramatically declined upon treatment with an FAK inhibitor, confirming the engagement of FAK in the regulation of phagocytosis in oyster hemocytes. According to the focal adhesion pathway, not only FAK was upregulated, but also its partners, integrin, filamin A, and P130cas, have high expression in phagocytes. Focal adhesion kinase as a cytoplasmic tyrosine kinase is typically activated by interaction with integrins at sites of focal contact, leading to reorganization of cytoskeleton with downstream factors, such as p130Cas and filamin A, to promote phagocytosis (62–64) (Figure 7). Interestingly, high FAK expression was also observed during the late-stage tumorigenesis in human, which implicates critical role of FAK in cancer progression and metastasis (65). Given that cancer cells and phagocytes share some common capacities such as high motility and infiltration, FAK signaling may be mechanistically exploited as a conserved pathway in those distinct cell models.

Quite strikingly, eight members of HSPGs are found to be lineage-specifically expanded and predominantly expressed in phagocytes, strongly supporting an important role of HSPG in activated phagocytes. Heparan sulfate proteoglycans are a family of typical proteoglycans with one or more covalently attached heparan sulfate (HS) chains (66, 67). Substantial studies show that HSPGs can act as versatile regulators in diverse signaling pathways, including Wnt, Hedgehog, and transforming growth factor β, thus impacting cell functions in development, cell migration, and autophagy (68–70). Moreover, HSPGs have been found to operate as cell surface receptors to mediate endocytosis or internalization (71, 72). As phagocytosis is one specific form of endocytosis, it is reasonable to speculate that HSPG may take part in the regulation of phagocytic function in oysters. Two chemical agents, chlorate and heparin, were used to verify the function of HSPGs in our study. Chlorate is a potent inhibitor of sulfation reactions in the biosynthesis of GAGs, which include HSs, an indispensable component for decoration of HSPGs (73, 74). Treatment of chlorate can effectively suppress the phagocytic activity in oyster hemocytes, corroborating the crucial roles of HSPGs as mentioned previously. Because chlorate can indiscriminately modulate sulfation of GAGs including HSs, chondroitin sulfate (CS), and dermatan sulfate (DS), we can't preclude the possibility that CS/DS proteoglycans may also be involved in chlorate-mediated phagocytic suppression (75). However, the use of heparin helps to establish the importance of HS in phagocytosis, as it can block the interactions of HS with its binding partners (76). Therefore, dose-dependent repressive effects of chlorate and heparin on the phagocytic activity in oyster hemocytes constitute evidence supporting the notion that HSPGs can function as cell surface receptors to mediate phagocytosis in invertebrates.

In mammals, it has been shown that infections by human papillomavirus type 16 depend on HSPG binding with viral particles and integrin-induced FAK activation (77). Moreover, an orthologous protein of HSPG (syndecan 4) was found to be necessary for focal adhesion formation and related downstream signal transduction (78). Taken together, these findings have shed light on the molecular details of phagocytic activation in oyster hemocytes (Figure 7). Elaborate matrices of HSPGs exist in high abundance in these phagocytes as cell surface receptors to capture invading bacteria and subsequently transduce danger signals by activating FAK signaling with the aid of integrins. Eventually, FAK signaling cascades trigger cytoskeletal remodeling to promote phagocytosis and other aspects of antibacterial defense. In addition, it has been reported that signaling pathways for cell aggregation are conserved and indispensable to phagocytosis in bivalve hemocytes (79). In this study, we observed that phagocytes manifested a higher aggregation rate compared to non-phagocytes. Pharmacological blockage of HSPGs synthesis strikingly changed the morphology of hemocytes and halted their aggregation in oysters, again shedding light on distinct aspects of HSPG function in regulating phagocyte behaviors.

In conclusion, an ingenious yet practically facile method was proposed to separate phagocytes and non-phagocytes efficiently. Transcriptomic analysis yielded fresh insights into the fundamental molecular differences between these groups. According to existing databases, there were ample DEGs contributing to their phenotypic disparity, of which we examined in detail two key factors, FAK and HSPGs. We then proceeded to experimentally verify their roles in phagocytic function. Both FAK and HSPGs showed significant effects on phagocytosis in hemocytes. Moreover, HSPG apparently also played a crucial role in hemocyte aggregation. Phagocytosis is an integral part of invertebrate innate immunity, requiring the cooperation of numerous sophisticated biomolecules, engineered to protect host cells. Based on our current transcriptomic analysis, a large number of such active biomolecules have been revealed, whose exact biological roles warrant further exploration or validation.

## Data Availability Statement

The datasets generated for this study can be found in the SRR10531303- SRR10531311.

## Author Contributions

YL implemented phagocyte sorting, flow cytometry detecting phagocytotic ability, and confocal microscopy imaging the phenomenon. YL, XZ, and KL analyzed the data of flow cytometry, while the fluorescence of pictures were calculated by ZX, JL, and SX. HM and MH performed RNA extraction for transcriptome. YZ and FM carried out bioinformatics analyses of transcriptomic data. YZ and ZY designed the research. YZ, YL, and N-KW wrote the manuscript and language proof.

### Conflict of Interest

The authors declare that the research was conducted in the absence of any commercial or financial relationships that could be construed as a potential conflict of interest.

## Supplementary Material

Supplementary Figure 1 The pictures of Phagocytes and Non-Phagocytes.
