# Supplementary material for: Phagocyte Transcriptomic Analysis Reveals Focal Adhesion Kinase (FAK) and Heparan Sulfate Proteoglycans (HSPGs) as Major Regulators in Anti-bacterial Defense of Crassostrea hongkongensis
Source: Front Immunol. 2020 Mar 20;11:416. doi: 10.3389/fimmu.2020.00416 (PMC7103635; doi:10.3389/fimmu.2020.00416)
Supplement: Supplementary file 1 [file Table_1.DOCX]

| **Sample name** | **Total Reads** | **Total Mapped Reads (%)** | **Unique Match (%)** | **Total Unmapped Reads (%)** | **Multi-position Match (%)** |
| --- | --- | --- | --- | --- | --- |
| **Hemocytes1** | 23700126 | 76.38 | 71.89 | 23.62 | 4.48 |
| **Hemocytes2** | 23661342 | 78.12 | 73.25 | 21.88 | 4.87 |
| **Hemocytes3** | 24071394 | 77.28 | 72.58 | 22.72 | 4.7 |
| **Non_Phagocyte1** | 24068277 | 76.98 | 71.63 | 23.02 | 5.35 |
| **Non_Phagocyte2** | 23785277 | 76.18 | 71.34 | 23.82 | 4.84 |
| **Non_Phagocyte3** | 23782438 | 76.6 | 71.75 | 23.4 | 4.85 |
| **Phagocyte1** | 23810696 | 79.62 | 73.82 | 20.38 | 5.8 |
| **Phagocyte2** | 23742312 | 80.19 | 74.53 | 19.81 | 5.65 |
| **Phagocyte3** | 24068212 | 79.75 | 74.22 | 20.25 | 5.53 |

**Table S1. Gene mapping of Hong kong oyster hemocyte samples.**
