# Supplementary material for: Phagocyte Transcriptomic Analysis Reveals Focal Adhesion Kinase (FAK) and Heparan Sulfate Proteoglycans (HSPGs) as Major Regulators in Anti-bacterial Defense of Crassostrea hongkongensis
Source: Front Immunol. 2020 Mar 20;11:416. doi: 10.3389/fimmu.2020.00416 (PMC7103635; doi:10.3389/fimmu.2020.00416)
Supplement: Supplementary file 4 [file Table_4.DOCX]

**Table S4. The accession numbers of HSPGs from Transcriptome and GenBank.**

| **Sample name** | **Accession numbers** |
| --- | --- |
| ChHSPGX1 | Unigene0002198 |
| ChHSPGX2 | Unigene0000860 |
| ChHSPGX3 | Unigene0000861 |
| ChHSPGX4 | Unigene0000859 |
| ChHSPGX5 | Unigene0002196 |
| ChHSPGX6 | Unigene0031082 |
| ChHSPGX7 | Unigene0002195 |
| ChHSPGX8 | Unigene0002197 |
| CgHSPGX5 | XP_019922929.1 |
| CgHSPGX3 | XP_019922927.1 |
| CgHSPGX6 | XP_019922930.1 |
| CgHSPGX4 | XP_019922928.1 |
| CgHSPGX2 | XP_019922926.1 |
| CgHSPGX1 | XP_011427908.1 |
| CgHSPG | EKC41388.1 |
| CvHSPGX7 | XP_022341963.1 |
| CvHSPGX6 | XP_022341962.1 |
| CvHSPGX5 | XP_022341961.1 |
| CvHSPGX4 | XP_022341960.1 |
| CvHSPGX3 | XP_022341959.1 |
| CvHSPGX2 | XP_022341958.1 |
| CvHSPGX1 | XP_022341957.1 |
| MyHSPGX3 | XP_021372608.1 |
| MyHSPGX1 | XP_021372605.1 |
| MyHSPGX7 | XP_021372612.1 |
| MyHSPGX4 | XP_021372609.1 |
| MyHSPGX6 | XP_021372611.1 |
| MyHSPG | OWF41229.1 |
| MyHSPGX2 | XP_021372607.1 |
| MyHSPGX8 | XP_021372613.1 |
| MyHSPGX5 | XP_021372610.1 |
| ObHSPGX4 | XP_014784088.1 |
| ObHSPGX | XP_014784085.1 |
| ObHSPGX1 | XP_014784092.1 |
| ObHSPGX6 | XP_014784091.1 |
| ObHSPGX5 | XP_014784090.1 |
| ObHSPGX3 | XP_014784087.1 |
| ObHSPGX2 | XP_014784086.1 |
| ObHSPGX8 | XP_019317238.1 |
