# Supplementary figures and images for: Phagocyte Transcriptomic Analysis Reveals Focal Adhesion Kinase (FAK) and Heparan Sulfate Proteoglycans (HSPGs) as Major Regulators in Anti-bacterial Defense of Crassostrea hongkongensis
Source: Front Immunol. 2020 Mar 20;11:416. doi: 10.3389/fimmu.2020.00416 (PMC7103635; doi:10.3389/fimmu.2020.00416)

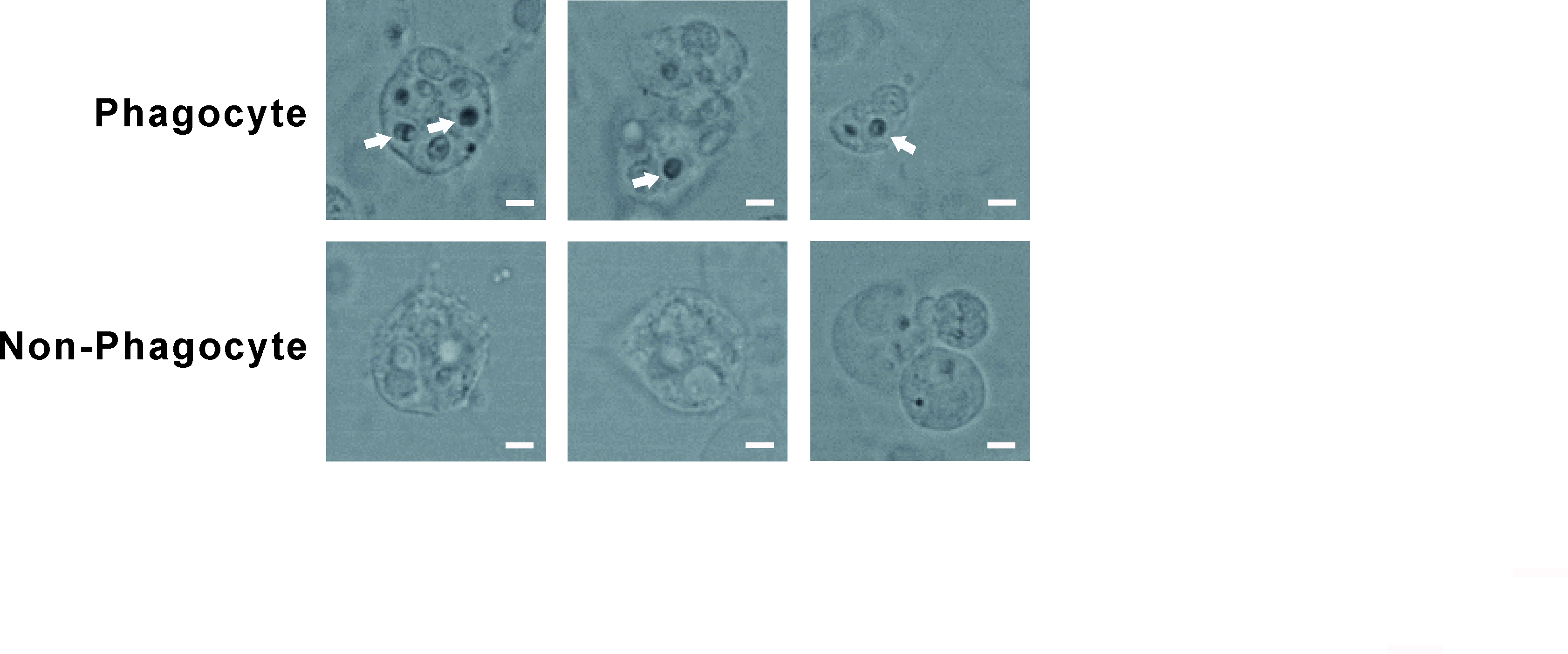

Supplement: Supplementary Figure 1 — The pictures of Phagocytes and Non-Phagocytes. [file Image_1.JPEG]
